# Temporal trends and current practice patterns for intraoperative ventilation at U.S. academic medical centers: a retrospective study

**DOI:** 10.1186/s12871-015-0010-3

**Published:** 2015-03-28

**Authors:** Jonathan P Wanderer, Jesse M Ehrenfeld, Richard H Epstein, Daryl J Kor, Raquel R Bartz, Ana Fernandez-Bustamante, Marcos F Vidal Melo, James M Blum

**Affiliations:** 1 Departments of Anesthesiology and Biomedical Informatics, Vanderbilt University, The Vanderbilt Clinic, 1301 Medical Center Drive, Suite 4648, Nashville, TN USA; 2 Departments of Anesthesiology, Biomedical Informatics, Health Policy and Surgery, Vanderbilt University, Nashville, TN USA; 3 Department of Anesthesiology, Sidney Kimmel College of Medicine at Thomas Jefferson University, Philadelphia, PA USA; 4 Department of Anesthesiology, Mayo Clinic, Rochester, MN USA; 5 Department of Anesthesiology, Duke University School of Medicine, Durham, NC USA; 6 Department of Anesthesiology, University of Colorado School of Medicine, Colorado, CO USA; 7 Department of Anesthesia, Critical Care and Pain Medicine, Massachusetts General Hospital, Boston, MA USA; 8 Department of Anesthesiology, Emory University Hospital, Atlanta, GA USA

**Keywords:** Intraoperative ventilation, Lung protective ventilation, Practice patterns

## Abstract

**Background:**

Lung protective ventilation strategies utilizing lower tidal volumes per predicted body weight (PBW) and positive end-expiratory pressure (PEEP) have been suggested to be beneficial in a variety of surgical populations. Recent clinical studies have used control groups ventilated with high tidal volumes without PEEP based on the assumption that this reflects current clinical practice. We hypothesized that ventilation strategies have changed over time, that most anesthetics in U.S. academic medical centers are currently performed with lower tidal volumes, and that most receive PEEP.

**Methods:**

Intraoperative data were pooled for adults undergoing general anesthesia with tracheal intubation. Median tidal volumes per kilogram of PBW were categorized as > 10, 8–10 and < 8 mL per kg of PBW. The percentages of cases in 2013 that were performed with median tidal volumes < 8 mL per kg of PBW and PEEP were determined. As a secondary analysis, a proportional odds model using institution, year, height, weight and gender determined the relative associations of these factors using categorical and interquartile odds ratios.

**Results:**

295,540 cases were analyzed from 5 institutions over a period of 10 years. In 2013, 59.3% of cases used median tidal volumes < 8 mL per kg of PBW, 83.3% used PEEP, and 51.0% used both. Of those cases with PEEP, 60.9% used a median pressure of ≥ 5 cmH_2_O. Predictors of lower categories of tidal volumes included height (odds ratio (OR) 10.83, 95% confidence interval [10.50, 11.16]), institution (lowest OR 0.98 [0.96, 1.00], highest OR 9.63 [9.41, 9.86]), year (lowest OR 1.32 [1.21, 1.44], highest OR 6.31 [5.84, 6.82]), male gender (OR 1.10 [1.07, 1.12]), and weight (OR 0.30 [0.29, 0.31]).

**Conclusion:**

Most general anesthetics with tracheal intubation at the institutions surveyed are currently performed with a median tidal volume < 8 mL per kg of PBW, most are managed with PEEP of ≥ 5 cmH_2_O and approximately half utilize both. Given the diversity of the institutions included, this is likely reflective of practice in U.S. academic medical centers. The utilization of higher tidal volumes without PEEP in control groups for clinical research studies should be reconsidered.

**Electronic supplementary material:**

The online version of this article (doi:10.1186/s12871-015-0010-3) contains supplementary material, which is available to authorized users.

## Background

The demonstration of a significant mortality benefit utilizing lung protective ventilation strategies in patients with acute respiratory distress syndrome (ARDS) suggests the possibility that using this approach may be beneficial during intraoperative ventilation in patients at risk for postoperative pulmonary complications [1]. Benefits from using lung protective ventilation strategies with tidal volumes below 8 mL per kilogram of predicted body weight (PBW) and positive end-expiratory pressure (PEEP) have been suggested in a variety of surgical populations [2-4], and could stem from reducing the risk of developing lung injury and other postoperative pulmonary complications. Higher tidal volumes that were historically used to prevent atelectasis (*e.g.*, greater than 10 mL per kg of PBW) are associated with lung inflammation in animal models [5-7], as well as worsened clinical outcomes in humans [8], which may be secondary to lung overinflation in at risk areas leading to systemic and pulmonary inflammatory responses [9,10]. While it is now generally accepted that lung protective ventilation is indicated for patients with established lung injury, it remains unclear what the most appropriate ventilation strategy is for patients with non-injured lungs at increased risk for postoperative pulmonary complications. Examples of such patients at risk include those with sepsis, cirrhosis, and those undergoing high-risk aortic vascular, high-risk cardiac surgery and emergency surgery [11]. Despite controversy [12] over whether available data are sufficient to support the use of lung protective ventilation in all surgical patients, there is evidence that clinical practice has already begun to adopt some elements of these approaches [13,14]. Importantly, these changes have potential implications for contemporary research regarding perioperative ventilator management in patients at increased risk for postoperative pulmonary complications.

Understanding the appropriate intraoperative ventilation strategy for patients requires experimental evidence that convincingly demonstrates improved outcomes in the relevant patient population compared to current recommendations. While there have been recent experimental studies with significant effects on meaningful outcomes, the control groups in those studies were ventilated with tidal volumes of 10–12 mL per kg of PBW without PEEP based on the assumption that such a strategy is routine in current clinical practice [3,15]. Indeed, a basic assumption of a major recent trial was that “use of high tidal volumes and no PEEP is still commonplace” [15]. Defining current clinical practices is a fundamental step in establishing appropriate control groups so that clinical research studies can be conducted in a clinically relevant context.

The purpose of this study is to elucidate temporal trends, and to determine current practice patterns in intraoperative ventilation to guide the design of future clinical research studies. We hypothesized that most adult, non-cardiothoracic and non-neurosurgical anesthetics from a sample at 5 academic medical centers in the U.S. are currently performed with median tidal volumes below 8 mL per kg of PBW. We further hypothesize that most of these cases received PEEP.

## Methods

### Patient population

This study received an exemption from institutional review board (IRB) approval by the Vanderbilt University Human Research Protection Program-Institutional Review Board, an exemption from IRB approval from the Colorado Multiple Institutional Review Board, IRB approval from Mayo Clinic’s Office for Human Research Protection-Institutional Review Board, IRB approval from Partners Human Research Committee, and a determination of non-human subject research from the Division of Human Subjects Protection at Thomas Jefferson University. At each institution, the requirement for written, informed consent from patients was waived. Patients aged 18 and older who received general anesthesia with tracheal intubation between 2005 and 2013 were screened. Each institution defined its own start date based on the availability of electronically captured ventilator data. Patients who underwent procedures with potential confounding ventilation considerations were not included, specifically those undergoing cardiac, thoracic, neurosurgical, laparoscopic and one lung ventilation procedures, as well as organ harvest. Cases without adequate data for analysis were excluded as described below.

### Data collection

Data were retrieved electronically from each institution’s anesthesia information management system (AIMS). At each site, AIMS data were extracted and de-identified. This was achieved by creating a de-identified case number, listing patients 90 years or older with an age of 90, removing date information with the exception of case year and calculating all physiologic and ventilator data timestamps relative to entry into the OR. Files were encrypted and transmitted using a secure website to Vanderbilt University, the coordinating institution, and were then loaded into a SQL Server database (Microsoft, Redmond, WA). The relevant data elements transmitted for each case were institution number, de-identified case number, year, American Society of Anesthesiologists (ASA) Physical Status, emergency *vs.* elective case status as defined by the presence of an ASA E flag, height in centimeters, weight in kilograms, age, gender, primary surgical service, surgical Current Procedural Terminology (CPT) code, anesthesia CPT code, total case duration (*i.e.*, entry to exit from room), times of surgical incision and end of surgery, use of lung isolation, use of a laparoscopic approach, and whether the case was a cardiac, thoracic or neurosurgical case. For each case, the following ventilation data were retrieved: peak inspiratory pressure, PEEP, exhaled tidal volume, mean airway pressure, respiratory rate (RR), ventilation mode, fraction of inspired oxygen (FiO_2_), oxygen saturation via pulse oximetry (SpO_2_) and end-tidal carbon dioxide (EtCO_2_). Not all data elements used to define the surgical procedure were available at all institutions; thus multiple data elements were included to define case exclusions.

### Data processing

**Figure 1:**
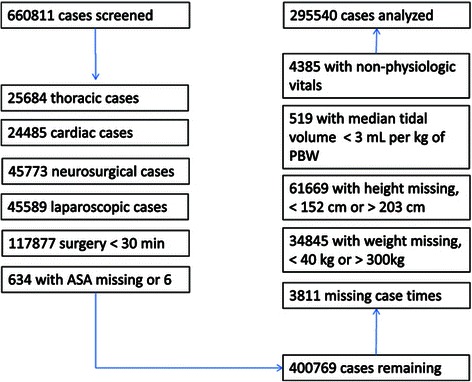
**Flow chart for cases included; A flow chart demonstrating the sequential exclusions applied to the initial set of cases, resulting in the cases included for analysis.** ASA = American Society of Anesthesiologists Physical Status Classification.

The minimum, maximum, average and median of each data element at each institution between incision and end of surgery were examined to rule out systematic problems with the data extraction process. The exclusion criteria were sequentially applied to the screened cases (Figure 1). Patients missing heights, or with a height less than 152 cm or greater than 203 cm were excluded to be consistent with prior work [13], as were those with unknown or missing genders, weight below 40 kg or above 300 kg, oxygen saturation (SpO2) above 100% or with median values below 50%, and median tidal volumes below 3 mL per kg of PBW. PBW was determined using gender and height per ARDSnet definition [16]. Specifically, for males, PBW was calculated as [(height (cm) -154) × 0.9] + 50. For females, PBW was calculated as [(height (cm) -154) × 0.9] + 45.5.

We excluded cases where there may have been overriding patient or procedural factors that may have altered routine ventilation strategies, such as one lung ventilation, cardiopulmonary bypass, elevated intracranial pressure, and use of laparoscopy. Specifically, we excluded thoracic, cardiac, and neurosurgical cases, and cases with use of laparoscopy. Thoracic cases were defined based on the primary surgical service field, a positive value for the one lung ventilation flag, or an anesthesia CPT code indicating one lung ventilation. Cardiac cases were defined based on the primary surgical service field. Neurosurgical cases were defined based on the primary surgical service field or an anesthesia CPT code indicating an intracranial procedure. Non-intracranial procedures performed by neurosurgical services were excluded. Laparoscopic cases were defined as those with a positive value for the laparoscopic flag or the presence of ‘laparoscopic’ at any point in the text description of the surgical procedure.

Surgical duration was calculated as the difference in minutes between the incision and end of surgery times. Where missing, the surgical end time was imputed as 30 minutes prior to leaving the OR. Cases with missing incision time were excluded. Brief cases (*i.e.,* surgical duration less than 30 minutes) were excluded to focus on the maintenance phase of anesthesia where the ventilation strategy is less likely to be impacted by preparation for extubation.

### Statistical analysis

Demographic variables were summarized with means and standard deviations for continuous variables and with percentages for categorical variables. Ventilator parameters were defined within each case at the 25^th^, 50^th^ and 75^th^ percentiles, restricting the analysis to the interval between incision and end of surgery. All tidal volumes refer to exhaled tidal volumes. Cases were divided into a three category ordered outcome with median tidal volumes > 10 mL per kg of PBW (traditional), 8–10 mL per kg of PBW (intermediate) and < 8 mL per kg of PBW (physiologic tidal volume during quiet breathing at rest), breakpoints selected to differentiate ventilation consistent with ARDSnet recommendations of 6–8 mL per kg of PBW [16] from traditional strategies of > 10 mL per kg used to prevent atelectasis [15], while also including an intermediate category. The primary analysis was to determine if most anesthetics performed in 2013 used a median tidal volume of < 8 mL per kg of PBW and if most of those anesthetics used PEEP. As a secondary analysis, a proportional odds model was used to examine factors associated with a higher tidal volume category (traditional *vs.* intermediate, intermediate *vs.* physiologic) using predictors previously identified [17] and adding institution and year. The pre-specified covariates were year, institution, gender, height and actual body weight. Body weight was modeled rather than body mass index, as height was already included in the model. As the number of included cases was large, height and body weight were modeled using restricted cubic splines with 4 knots (placed at quartiles 0.05, 0.35, 0.65, 0.95) in order to identify non-linear effects. The remaining parameters were modeled as categorical variables. The odds of receiving a lower category of tidal volume (traditional *vs.* intermediate, intermediate *vs.* physiologic) were computed for height and weight as interquartile odds ratios, which represent comparisons between the 25^th^ and 75^th^ percentile values for those parameters. Similarly, odds ratios were computed for categorical variables, using female gender, the year 2005 and institution A as references. We determined the mean values of all ventilation parameters on a per-year, per-institution basis, in addition to a per-year basis for all institutions for tidal volume and PEEP. Tidal volume per mL of actual body weight and per mL of PBW were compared. Two-tailed, independent t-tests were used for comparison of continuous variables. Data are presented with means and standard deviations, except where otherwise noted. The significance of differences between proportions were compared using z-ratios with two-tailed, independent binomial proportions tests. Statistical inference was performed using a significance level of 0.05. All analyses were performed using R (R Foundation for Statistical Computing, Vienna, Austria).

## Results

There were a total of 660,811 adult cases with general anesthesia and tracheal tube placement identified by the collaborating institutions. Of those cases, 400,769 (60.6%) were not excluded by design. Of these patients, 105,229 (26.2%) were excluded due to missing or excluded data, with 295,540 remaining for analysis. Details of the excluded cases are found in Figure 1. The distributions of age, gender, height and weight are noted in Table 1, with an overall mean age of 54 ± 16.5 years, a height of 170.3 ± 9.9 cm, a weight of 84 ± 22.2 kg and a body mass index of 28.9 ± 7.0 kg/m^2^. 95.7% of the analyzed cases were elective with a mean duration of 171 ± 119.4 minutes.

**Table 1:** **Data presented are counts or median values with 25th and 75th percentiles**

	Total	Center A	Center B	Center C	Center D	Center E
	(n= 296882)	(n=56740)	(n=108776)	(n=10833)	(n= 58156)	(n= 62377)
ASA						
1	26287	5825	8693	1245	2118	8406
2	151819	23839	62195	5042	23328	37415
3	108011	24164	35623	3766	28919	15539
4	10590	2889	2217	741	3747	996
5	175	23	48	39	44	21
Emergent case						
Yes	284121	53058	105515	9862	55868	59818
No	12761	3682	3261	971	2288	2559
Height	117 (162, 177)	117 (162,177)	170 (163, 177)	170 (162, 177)	172 (163, 180)	170 (162, 177)
Weight	81 (68, 96)	79 (66, 94)	82 (68, 97)	79 (67, 94)	83 (70, 99)	79 (66, 93)
Age	56 (43, 67)	54 (42, 66)	59 (46, 69)	52 (39, 63)	54 (42, 64)	55 (42, 66)
Duration	141 (89, 218)	111 (69, 187)	172 (118, 252)	108 (64, 178)	127 (78, 194)	129 (84, 205)
Male gender	49.18%	47.93%	48.85%	47.92%	52.28%	48.23%

ASA = American Society of Anesthesiologists Physical Status Classification.

### Current ventilation approach

There were 58,613 included cases performed during 2013. The median tidal volume for these cases was 7.8 ± 1.5 mL per kg of PBW. 34,751 (59.3%) cases had a median tidal volume less than or equal to 8 mL per kg of PBW. Of the included 2013 cases, 48,808 (83.3%) utilized PEEP with a median value of 5 cm H_2_O (25^th^ percentile 4, 75^th^ percentile 5). The overall percentage of cases performed in 2013 by PEEP category was 16.5% (none), 22.6% (0–4 cmH_2_O), 60.7% (5–10 cmH_2_O), and 0.2% (>10 cmH_2_O). There were 29,918 (51.0%) cases that had a median tidal volume less than or equal to 8 mL per kg of PBW that also utilized PEEP. As described below, in 2013 all centers (A, B, C, D, E) used PEEP for most cases (58.2%, 99.9%, 92.4%, 72.9%, 79.1% respectively *vs.* 50%, p < 0.0001), 3 of 5 centers used a median tidal volume less than or equal to 8 mL per kg of PBW for most cases (40.4%, 77.7%, 61.2%, 45.6%, 51.7% respectively *vs.* 50%, p < 0.0001), and 2 of 5 centers used a median tidal volume less than or equal to 8 mL per kg of PBW and PEEP for most cases (22.7%, 77.6%, 55.4%, 34.4%, 38.5% respectively *vs.* 50%, p < 0.0001).

### Tidal volumes

**Figure 2:**
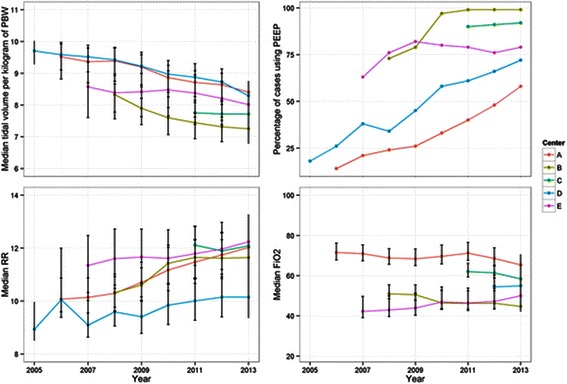
**Ventilation parameters set by the anesthesia provider; Ventilation parameters set by the anesthesia provider over time (2005–2013) at five U.S. academic medical centers.** At top left, average of median exhaled tidal volume per kilogram of predicted body weight (PBW). At top right, percentage of cases utilizing positive end expiratory pressure (PEEP). At bottom left, average of median respiratory rate (RR). At bottom right, average of median fraction of inspired oxygen (FiO_2_). Error bars indicate 25^th^ and 75^th^ percentile ranges.

**Figure 3:**
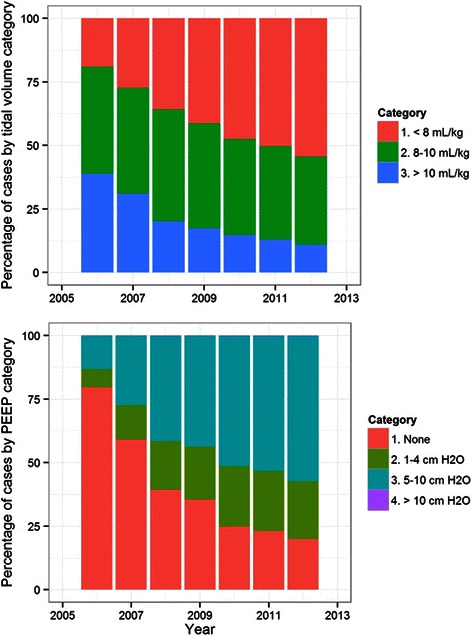
**Tidal volumes and PEEP; Median exhaled tidal volumes were grouped into categories of < 8 mL per kg of predicted body weight (PBW), 8–10 mL per kg of PBW and > 10 mL per kg of PBW.** Median positive end-expiratory pressure (PEEP) values were grouped into categories of no PEEP, 1–4 cm H_2_O, 5–10 cm H_2_O and > 10 cm H_2_O. Counts of cases with tidal volumes and PEEP usage are displayed above for each year analyzed.

The median tidal volume mL per kg of PBW was computed for each case, and the means of these tidal volumes were computed for each center and year (Figure 2, top left). Median tidal volume decreased from an average of 9.7 ± 1.9 mL per kg of PBW in 2005 to 7.8 ± 1.5 mL per kg of PBW in 2013 overall (p < 0.0001). Comparing the proportion of cases using a median tidal volume of < 8 mL per kg of PBW between the first and last year of data available at each center revealed an increasing use of median tidal volumes < 8 mL per kg of PBW at centers A (19.6% in 2006 *vs.* 40.4% in 2013, p < 0.0001), B (45.6% in 2008 *vs.* 77.7% in 2013, p < 0.0001), D (16.0% in 2005 *vs.* 45.6% in 2013, p < 0.0001) and E (39.4% in 2006 *vs.* 51.7% in 2013, p < 0.0001). No statistically significant difference was noted at center C (59.6% in 2011 *vs.* 61.2% in 2013, p = 0.3), although limited trend data were available at this center. Of all the cases analyzed, 43,934 (14.9%) had a median tidal volume of > 10 mL per kg of PBW, 110,207 (37.3%) had a median tidal volume of 8–10 mL per kg of PBW, and 141,463 had a median tidal volume of < 8 mL per kg of PBW (47.8%), with median tidal volume decreasing over time (Figure 3, top).

### Positive end-expiratory pressure

The utilization of PEEP was determined for each case, and the percentage of cases utilizing PEEP was computed for each center and year (Figure 2, upper right). Utilization of PEEP increased from 18.3% to 83.3% (p < 0.0001) over the study period, and was used with a median pressure of 5 cm H_2_O (4, 5) as seen in Figure 3. Comparing the proportion of cases using PEEP between the first and last year of data available at each center revealed an increasing use of PEEP at centers A (14.8% in 2006 *vs.* 58.2% in 2013, p < 0.0001), B (73.9% in 2008 *vs.* 99.9% in 2013, p < 0.0001), D (22.4% in 2005 *vs.* 72.9% in 2013, p < 0.0001) and E (63.0% in 2006 *vs.* 79.1% in 2013, p < 0.0001). No statistically significant difference was noted at center C (90.7% in 2011 *vs.* 92.4% in 2013, p = 0.056).

### Additional ventilation parameters

**Figure 4:**
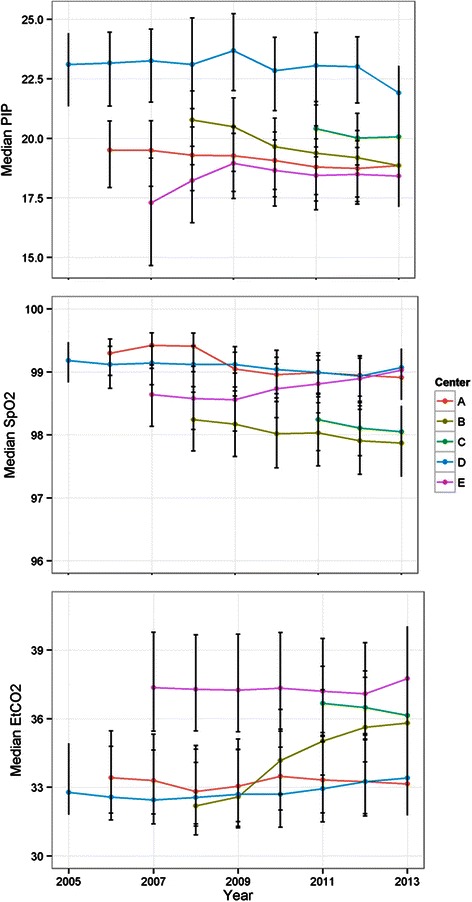
**Physiologic parameters; Physiologic parameters resulting from ventilation strategies (2005–2013) at five U.S. academic medical centers.** Average of median peak inspiratory pressure (PIP) at top, average of median blood oxygen saturation (SpO_2_) in middle, and average of median end-tidal carbon dioxide (EtCO_2_) at bottom. Error bars indicate 25^th^ and 75^th^ percentile ranges.

The median respiratory rate was computed for each case, and the means of these values were computed for each center and year (Figure 2, lower left). Median respiratory rate increased significantly over the study period from 8.9 in 2005 breaths per minute to 11.7 breaths per minute in 2013 (p < 0.0001). FiO2 utilization varied by institution (Figure 2, lower right), but did not result in clinically significant differences in SpO2 (Figure 4, middle). Trends of EtCO2 and peak inspiratory pressure similarly demonstrated institutional variation (Figure 4, bottom and top).

### Predictors of lower tidal volumes

**Figure 5:**
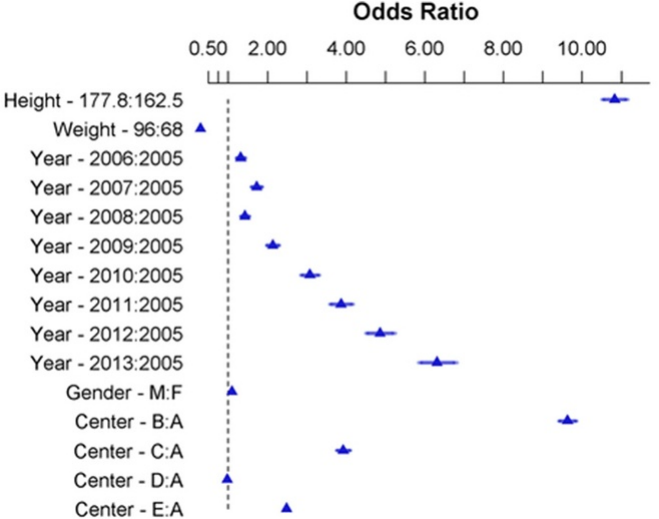
**Proportional odds model estimates; The proportional odds model estimates of the odds of receiving lower tidal volume ventilation for each variable compared to a reference value, with median exhaled tidal volume categorized as > 10 mL per kg of predicted body weight (PBW), 8–10 mL per kg of PBW, and < 8 mL per kg of PBW.** The triangles mark the estimate centers, with 95% confidence intervals indicated with the bars extending from the centers. For the categorical variables of year, gender and center, the estimates provided are odds of receiving lower tidal volume ventilation relative to 2005, females and center A, respectively. For height and weight, the estimates provided are the interquartile odd ratio, indicating the odds of receiving lower tidal volume ventilation at the 75^th^ percentile of those variables compared to the 25^th^ percentile. The p-value for each estimate is p < 0.0001 based on a likelihood ratio test with 11 degrees of freedom.

All of the pre-specified factors were significantly related to the tidal volumes utilized (Figure 5). The R^2^ for the proportional odds model was 0.470. Height had the largest odds ratio for receiving lower tidal volumes (odds ratio (OR) 10.83, 95% confidence interval [10.50, 11.16]), followed by institution (lowest OR 0.98 [0.96, 1.00], highest 9.63 [9.41, 9.86]), year (lowest OR 1.32 [1.21, 1.44], highest OR 6.31 [5.84, 6.82]), male gender (OR 1.10 [1.07, 1.12]), and weight (OR 0.30 [0.29, 0.31]). Modeling with restricted spline curves allowed for visualization of non-linear relationships between the probability of receiving lower tidal volume ventilation and each previously identified risk factor for higher tidal volume ventilation; no specific cut-off for increased risk was identified for weight or height (Additional file 1). Factor associated with higher tidal volumes included female gender, increased body weight, decreased stature and cases performed in earlier years. Significant differences were also noted by institution, which varied from an OR of 0.98 (0.96, 1.00) for institution D compared with institution A, to an OR of 9.63 (9.41, 9.86) for institution B compared with institution A.

### Comparison of actual and predicted body weight

The overall mean for median tidal volumes across all institutions and years was 8.3 ± 1.8 mL per kg of PBW, compared to an overall mean of 6.3 ± 1.6 mL per kg of actual body weight (Additional file 2). This difference was statistically significant (p < 0.0001).

## Discussion

We confirmed our hypothesis that most (59.3%) anesthetics are currently being performed with tidal volumes below 8 mL per kg of PBW in U.S. academic medical centers. We further confirmed that most (83.3%) cases received PEEP. Approximately half (51%) of current anesthetics utilized both tidal volumes below 8 mL per kg of PBW and PEEP. This analysis has revealed a significant trend toward decreased tidal volumes relative to PBW and an increased utilization of PEEP, with a rise in respiratory rate. The previously identified predictors [17] for non-compliance with lower tidal volume ventilation including decreased height, female gender and higher body weight were validated, and significant effects from time and institution were noted.

It is possible that the changes we observed in the intraoperative period are a reflection of broader changes in ventilation strategy both outside and inside of the operating room. Following the ARDSnet trial which demonstrated a mortality benefit from decreased tidal volumes and the concomitant application of PEEP to ICU patients with established lung injury, protective lung ventilation has gained increasing acceptance. While historically higher tidal volumes (>10 mL per kg of PBW) were chosen to prevent atelectasis, data from animal models [5-7] and humans [8] have shown an increase in inflammatory markers associated with higher tidal volumes used without PEEP, although these effects have not been consistently observed [18].

A recent randomized controlled trial of intraoperative ventilation strategies conducted in France demonstrated a mortality benefit when using lower tidal volumes (6–8 mL per kg of PBW) with PEEP compared to a control group that utilized higher tidal volumes (10–12 mL per kg of PBW) without PEEP [15]. However, as our data demonstrate, the ventilation strategy that the control group received in that trial does not reflect the current practices for intraoperative ventilation in major U.S. academic medical centers. Our findings indicate that the most patients in major U.S. academic medical centers are currently receiving intraoperative ventilation strategies consistent with protective lung ventilation with respect to lower tidal volumes and usage of PEEP, although our findings also demonstrate that a large proportion of these patients are not receiving both, and a significant number of patients receive neither. While it is premature to conclude that intraoperative protective lung ventilation strategies may be indicated for patients undergoing surgical procedures, there is a growing body of evidence to suggest that certain patient conditions and surgical procedures are associated with higher incidences of postoperative pulmonary complications [19].

While the observed trend to lower tidal volumes and consistent use of PEEP likely reflects the increased awareness of the benefits of minimizing mechanical injury to the lungs, the exact settings to achieve that goal within the operative context are largely unknown. Direct application of ICU findings to the OR setting is probably unsuitable given the mechanical differences between injured and non-injured lungs, in addition to the potential influence of inflammatory mediators and ongoing lung injury from infection. Although surgical inflammation may predispose the lungs to injury, this hypothesis is untested. Accordingly, additional research using control groups that reflect current practice is needed to determine optimal intraoperative ventilation strategies.

Our finding of substantial variability amongst the centers included in this study indicates a lack of clear evidence and guidelines to precisely direct clinical use of intraoperative mechanical ventilation. It further implies that whereas a global trend exists for ventilation strategies, the specific ventilator settings used are also a matter of local practice, even in centers where the best scientific information is readily available. Together, these findings suggest that not only is better evidence needed to help guide clinical practice, but also that education will be an essential component in the effort to implement best ventilator practice guidelines at all levels.

Our study has a number of limitations. We examined only U.S. academic medical centers. Consequently, we are unable to determine if the current practice patterns observed are applicable in community hospitals, same-day surgical centers or other settings where intraoperative mechanical ventilation occurs. However, as these centers are representative of our nation’s training pipeline, it is likely that trainees, including anesthesiology residents and student certified registered nurse anesthetists, who graduate from these programs follow similar practices. Additionally, we excluded procedures where there might be additional, procedure-related considerations in choosing a ventilation strategy, specifically neurosurgical procedures, cardiovascular procedures and procedures using laparoscopy. Our conclusions thus do not generalize to those procedures. We further excluded pediatric patients, limiting our analysis to adult patients. We studied only patients who underwent general anesthesia with tracheal intubation and so are not able to determine what trends may or may not have occurred with other airway approaches such as supraglottic airway devices. As our data were comprised of millions of data points across many institutions there are likely data artifacts that remained present despite best efforts to validate these data and to exclude cases with any invalid data. These data quality concerns were mitigated by choosing statistical analysis techniques that are robust with respect to artifacts that manifest themselves in the extremes of data values. Additionally, we limited the analysis to cases that were at least 30 minutes in length with corresponding automated measurements of tidal volume, thus focusing our study on the maintenance phase of anesthesia where the ventilation strategy is less likely to be impacted by preparation for extubation. Finally, our study focused on tidal volume and PEEP only, and did not examine in detail other aspects of ventilator management that could be considered part of lung protective ventilation, including FiO2, peak inspiratory pressure, plateau pressure and mean airway pressure.

## Conclusion

In conclusion, we have analyzed the intraoperative ventilation approach from a sample of adult, non-cardiothoracic and non-neurosurgical anesthetics from U.S. academic medical centers, which was significant for a trend towards increasing usage of PEEP and decreasing tidal volume over time. Most anesthetics in these institutions are currently conducted with tidal volumes of less than 8 mL per kg of PBW, most anesthetics are also performed with PEEP and approximately half are performed with both.

## Additional files

Additional file 1: **Proportional odds model results expressed as predicted probability of receiving a lower tidal volume category, with median exhaled tidal volumes categorized as > 10 mL per kg of predicted body weight (PBW), 8-10 mL per kg of PBW, and < 8 mL per kg of PBW.** Height and weight were modeled using restricted spline curves with 4 knots (at quantiles 0.05, 0.35, 0.65, 0.95). The solid blue lines shows the estimated probability of receiving lower tidal volumes, and the grey bands and bars show the lower and upper 95% confidence interval of those estimates for continuous and categorical variables, respectively. Additional file 2: **Tidal volume comparison of actual versus predicted body weight; A comparison of tidal volumes per mL of actual body weight (red) and tidal volumes per mL of predicted body weight (blue).** This distribution includes median exhaled tidal volumes from all institutions and study years.

## Abbreviations

ARDS: Acute respiratory distress syndrome
ASA: American society of anesthesiologists
AIMS: Anesthesia information management system
CPT: Current procedural terminology
EtCO2: End-tidal carbon dioxide
FiO2: Fraction of inspired oxygen
PBW: Predicted body weight
PEEP: Positive end-expiratory pressure
RR: Respiratory rate
OR: Odds ratio
SpO2: Oxygen saturation
