# Supplementary figures and images for: Temporal trends and current practice patterns for intraoperative ventilation at U.S. academic medical centers: a retrospective study
Source: BMC Anesthesiol. 2015 Mar 28;15:40. doi: 10.1186/s12871-015-0010-3 (PMC4387596; doi:10.1186/s12871-015-0010-3)

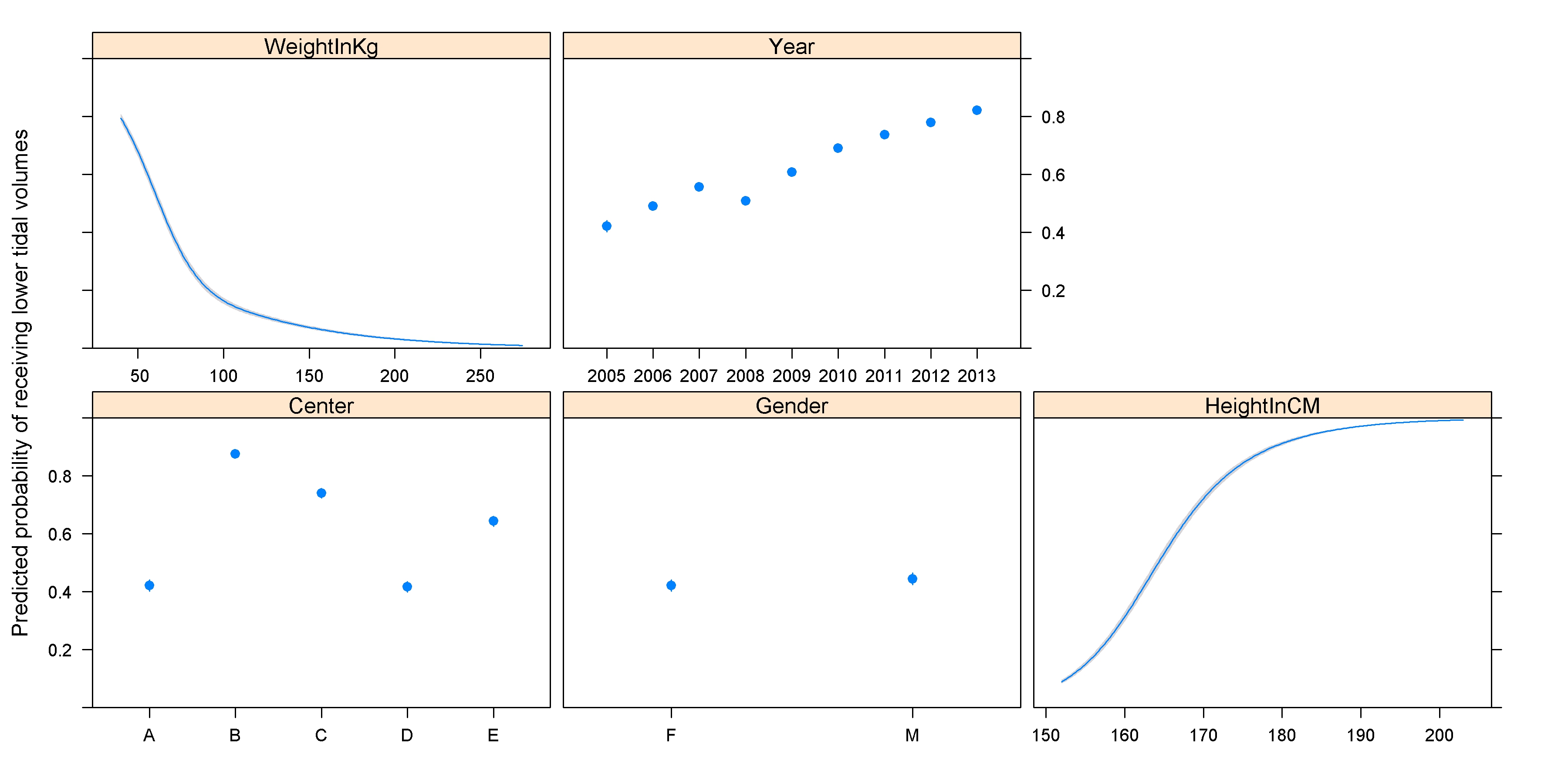

Supplement: Additional file 1: — Proportional odds model results expressed as predicted probability of receiving a lower tidal volume category, with median exhaled tidal volumes categorized as > 10 mL per kg of predicted body weight (PBW), 8-10 mL per kg of PBW, and < 8 mL per kg of PBW. Height and weight were modeled using restricted spline curves with 4 knots (at quantiles 0.05, 0.35, 0.65, 0.95). The solid blue lines shows the estimated probability of receiving lower tidal volumes, and the grey bands and bars show the lower and upper 95% confidence interval of those estimates for continuous and categorical variables, respectively. [file 12871_2015_10_MOESM1_ESM.jpg]

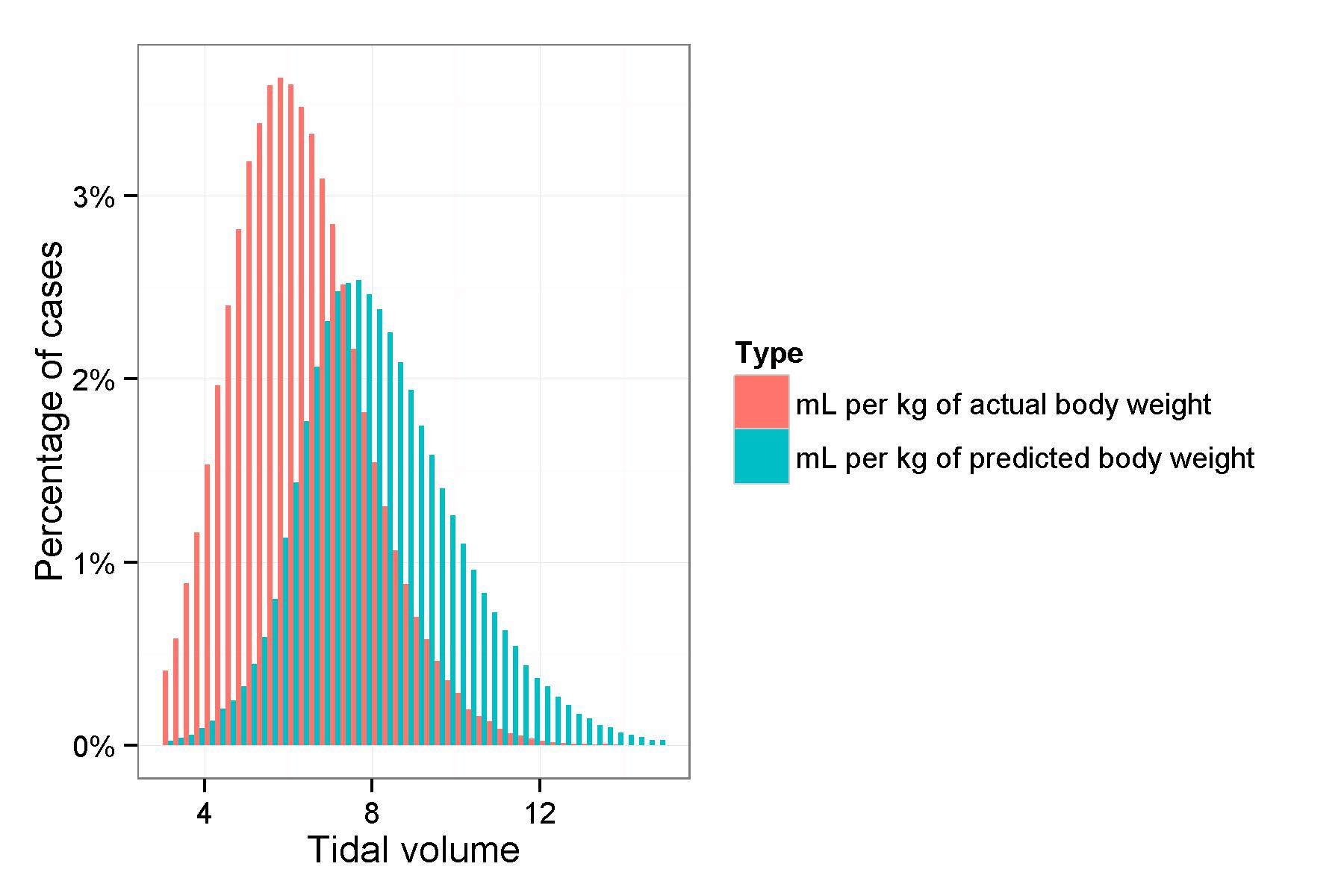

Supplement: Additional file 2: — Tidal volume comparison of actual versus predicted body weight; A comparison of tidal volumes per mL of actual body weight (red) and tidal volumes per mL of predicted body weight (blue). This distribution includes median exhaled tidal volumes from all institutions and study years. [file 12871_2015_10_MOESM2_ESM.jpg]
